# Colchicine inhibits ROS generation in response to glycoprotein VI stimulation

**DOI:** 10.1038/s41598-021-91409-7

**Published:** 2021-06-07

**Authors:** G. J. Pennings, C. J. Reddel, M. Traini, H. Campbell, V. Chen, L. Kritharides

**Affiliations:** 1 The ANZAC Research Institute, Concord Repatriation General Hospital, Hospital Road, Concord, NSW 2139 Australia; 2 Department of Haematology, Concord Repatriation General Hospital, Concord, NSW Australia; 3 Department of Cardiology, Concord Repatriation General Hospital, Hospital Road, Concord, NSW 2139 Australia; 4 The University of Sydney, Sydney, NSW Australia

**Keywords:** Cell biology, Medical research

## Abstract

Colchicine inhibits coronary and cerebrovascular events in patients with coronary artery disease (CAD), and although known to have anti-inflammatory properties, its mechanisms of action are incompletely understood. In this study, we investigated the effects of colchicine on platelet activation with a particular focus on its effects on activation via the collagen glycoprotein (GP)VI receptor, P2Y_12_ receptor, and procoagulant platelet formation. Therapeutic concentrations of colchicine in vitro (equivalent to plasma levels) significantly decreased platelet aggregation in whole blood and in platelet rich plasma in response to collagen (multiplate aggregometry) and reduced reactive oxygen species (ROS) generation (H_2_DCF-DA, flow cytometry) in response to GPVI stimulation with collagen related peptide-XL (CRP-XL, GPVI specific agonist). Other platelet activation pathways including P-selectin expression, GPIIb/IIIa conformational change and procoagulant platelet formation (GSAO^+^/CD62P^+^) (flow cytometry) were inhibited with higher concentrations of colchicine known to inhibit microtubule depolymerization. Pathway specific mechanisms of action of colchicine on platelets, including modulation of the GPVI receptor pathway at low concentrations, may contribute to its protective role in CAD.

## Introduction

Colchicine is well known as an anti-inflammatory drug used in the treatment of acute gouty arthritis, familial Mediterranean fever and Behçet’s disease^1^. Additionally, it has been used for the treatment of pericarditis^2^, atrial fibrillation post-coronary bypass, stroke and for secondary prevention of cardiac events^3–7^. More recently it has been investigated in the setting of coronary artery disease (CAD)^8–11^. In the laboratory, nanomolar (typical plasma levels, 1.25–21 nM^12,13^) and millimolar (known to inhibit microtubule formation^14,15^) concentrations of colchicine exert distinct anti-inflammatory effects suggesting a range of mechanisms are at play^16–18^.

Platelets have a key role in the development and progression of CAD^19,20^, but little is known about the effect of colchicine on platelet function. Early studies demonstrated a colchicine concentration-dependent decrease in aggregation in response to ADP, epinephrine and collagen in vitro^21–26^. Serotonin release in response to Ca^2+^ ionophore A23187 was also inhibited by colchicine^25^. A more recent in vivo study in healthy controls observed that colchicine decreased platelet-neutrophil aggregates, P-selectin expression in response to thrombin and decreased conformational change of GPIIb/IIIa in response to epinephrine but did not inhibit platelet aggregation in vitro unless supratherapeutic levels of colchicine were used^27^. In another study, colchicine (10 µM) suppression of platelet aggregation in response to various platelet agonists was in part a consequence of microtubule depolymerisation and inhibition of key proteins involved in cytoskeleton rearrangement^28^. The same group reported that higher concentrations of colchicine suppressed aggregation in response to stimulation with ADP in patients receiving dual antiplatelet therapy and in clopidogrel non-responders^29^. We recently demonstrated that colchicine attenuated the release of platelet extracellular vesicles and platelet activation in a microtubule-dependent manner and decreased procoagulant activity (in plasma) in response to mild platelet activation and traditional platelet agonists (ADP and epinephrine)^30^.

Contemporary understanding of platelet activation pathways in relation to cardiovascular disease and thrombosis, including important roles for collagen-dependent activation via glycoprotein (GP)VI, P-selectin^31^, GPIIb/IIIa conformational activation^32^ and the formation of prothrombotic 4-(*N*-(*S*-glutathionylacetyl)amino) phenylarsonous acid (GSAO)-binding platelets^33,34^, have emerged since the original colchicine studies on platelets were performed. Novel platelet pathways may contribute to the recent favourable cardiovascular outcome studies with colchicine (LoDoCO2^11^) which have in some respects exceeded the benefits achieved with targeted IL-1β antagonism (CANTOS^35^), suggesting mechanisms in addition to the inhibition of inflammasomal activity in leukocytes. For example, colchicine caused a reduction in soluble GPVI, a factor that is only released from platelets^11^.

Collagen receptors (e.g., GPVI) and purinergic receptors (e.g., P2Y_12_) are important but distinctive pathways in platelet activation and aggregation. Binding of collagen or an appropriate ligand to GPVI leads to protein phosphorylation (e.g. Syk and PLCγ2) and subsequent reactive oxygen species (ROS) generation, intracellular calcium flux, activation and conformational change of GPIIb/IIIa (reviewed in^36^). Binding of ADP to P2Y_12_ also leads to platelet activation in response to intracellular calcium changes and conformational change of GPIIb/IIIa^37^. GPVI is also a novel antiplatelet target for the treatment of inflammatory disorders including CAD (reviewed in^36,38,39^), and levels of membrane bound and soluble GPVI predict outcomes after myocardial infarction and stroke (reviewed in^40^). GPVI is stimulated by collagen released or exposed from within the subendothelial matrix upon injury to the endothelium and by fibrinogen and fibrin that is present during thrombus formation, among other ligands^39^. Recent studies have identified that anti-GPVI therapy can lead to disaggregation of thrombus in the absence of thrombin^41^.

We investigated the effect of colchicine, at therapeutic plasma concentration and at microtubule depolymerisation concentration, on platelet aggregation in response to collagen and ADP, and the downstream effects of colchicine after GPVI and P2Y_12_ stimulation on ROS generation and calcium flux, respectively. Additionally, we examined the in vitro effects of colchicine on platelet activation and procoagulant platelet formation.

## Results

### Colchicine inhibits platelet aggregation

**Figure 1:**
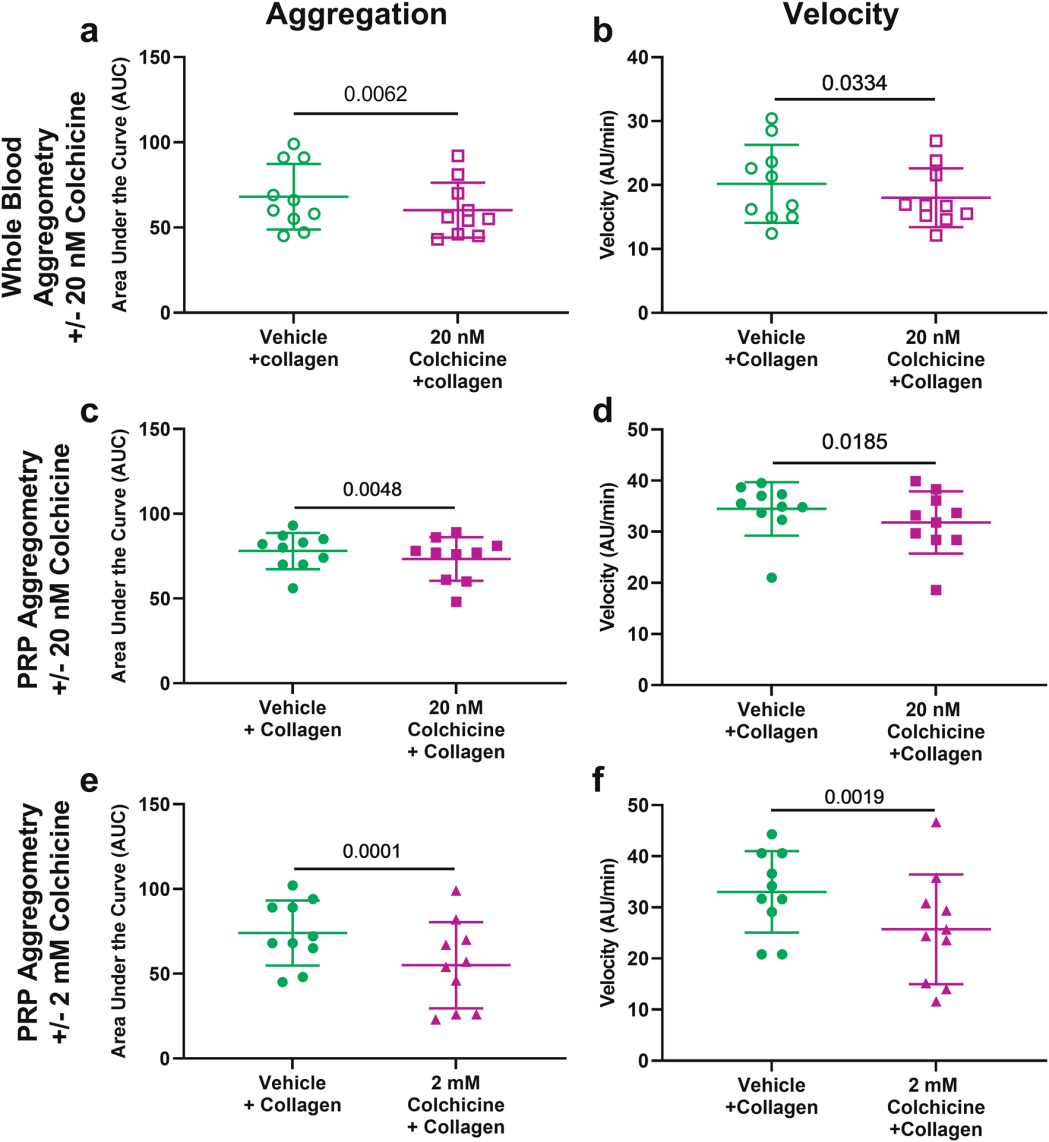
Colchicine decreases platelet aggregation response to collagen in whole blood and platelet rich plasma (PRP). Hirudinised whole blood and PRP from healthy controls (n = 10) was assessed for platelet aggregation and the rate of aggregation (velocity) after incubation with collagen (3 µg/mL) in the presence or absence of 20 nM colchicine and 2 mM colchicine (PRP only). Significant decrease in aggregation and velocity was observed in whole blood (p = 0.006 and p = 0.03; **a** and **b**) and in PRP after incubation with 20 nM colchicine (p = 0.005 and p = 0.019; **c** and **d**) and 2 mM colchicine (p = 0.0001 and 0.0019; **e** and **f**). Statistical significance was determined by paired *t* test to vehicle control. Data presented as mean ± SD.

**Figure 2:**
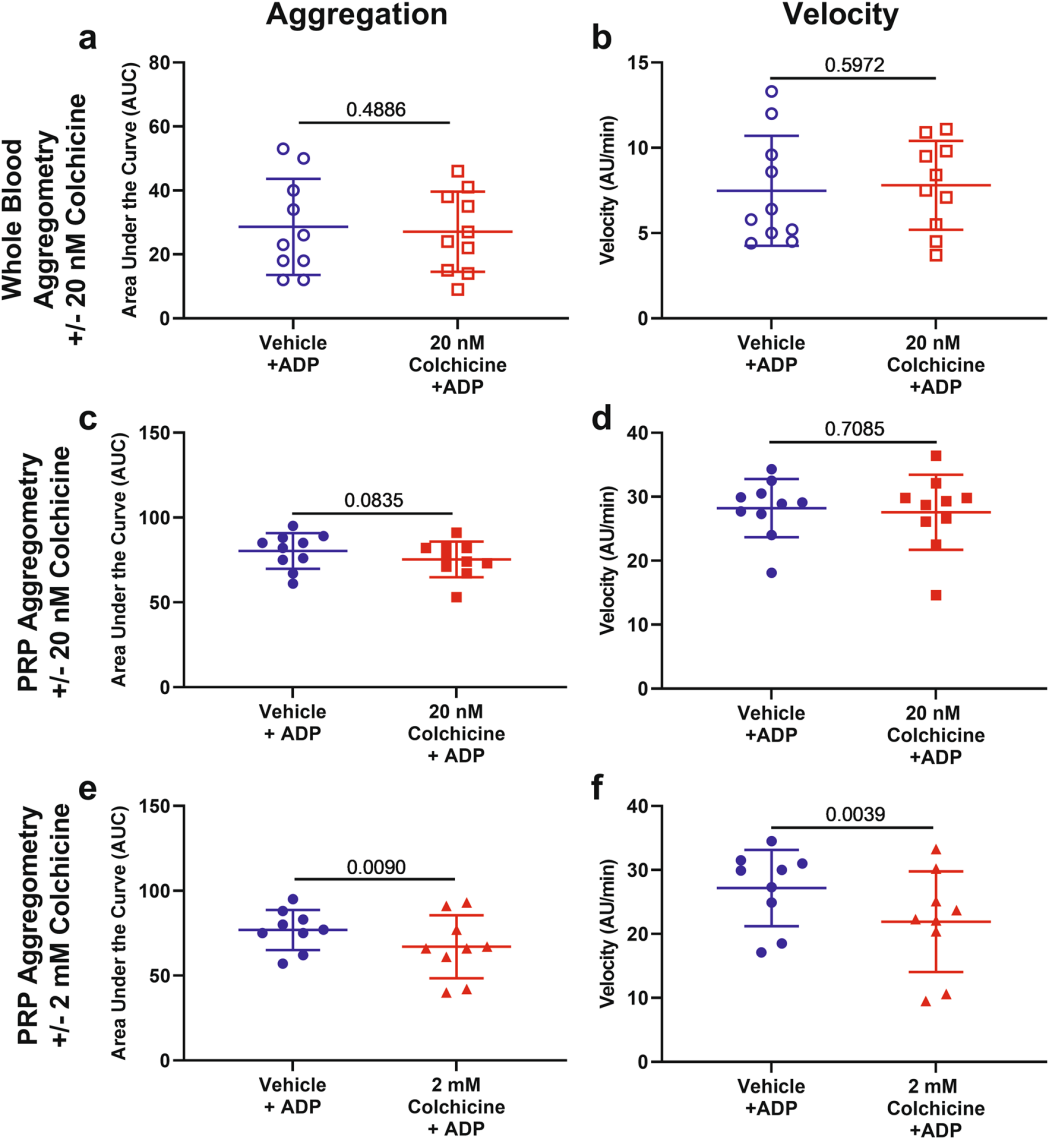
Colchicine decreases ADP-induced platelet aggregation in platelet rich plasma (PRP). Hirudinised whole blood and PRP from healthy controls (n = 9–10) was assessed for platelet aggregation and the rate of aggregation (velocity) after incubation with ADP (6.7 µM) in the presence or absence of 20 nM colchicine and 2 mM colchicine (PRP only). No significant decrease in aggregation or velocity was observed in whole blood (**a**,**b**) or in PRP after incubation with 20 nM colchicine (**c**,**d**). Significant decrease in aggregation and velocity was observed in PRP after incubation with 2 mM colchicine (p = 0.009 and 0.004; **e**,**f**). Statistical significance was determined by paired *t* test to vehicle control. Data presented as mean ± SD.

Colchicine pre-incubation significantly inhibited platelet aggregation in response to stimulation with 3 µg/mL collagen in both whole blood (20 nM colchicine, n = 10, p = 0.006, Fig. 1a) and platelet-rich plasma (PRP, n = 10, p = 0.005, Fig. 1c). The rate of aggregation was also significantly affected by 20 nM colchicine (whole blood, p = 0.03, Fig. 1b and PRP, p = 0.02, Fig. 1d) and 2 mM colchicine (p = 0.002, Fig. 1f). At 2 mM colchicine, the inhibition of platelet aggregation in response to collagen was over 25% (74.00 ± 19.17 vs 55.00 ± 25.44 AUC, n = 10, p = 0.0001, Fig. 1e). Colchicine (20 nM) did not affect platelet aggregation or velocity in response to 6.7 µM ADP in whole blood or PRP (Fig. 2a–d). However, 2 mM colchicine did decrease platelet aggregation in PRP in response to ADP (p = 0.009, Fig. 2e) and the rate of aggregation was also reduced (p = 0.0039, Fig. 2f). Colchicine inhibited collagen and CRP-XL induced aggregation to a similar degree (Suppl Fig. 1).

### Colchicine inhibits ROS generation

**Figure 3:**
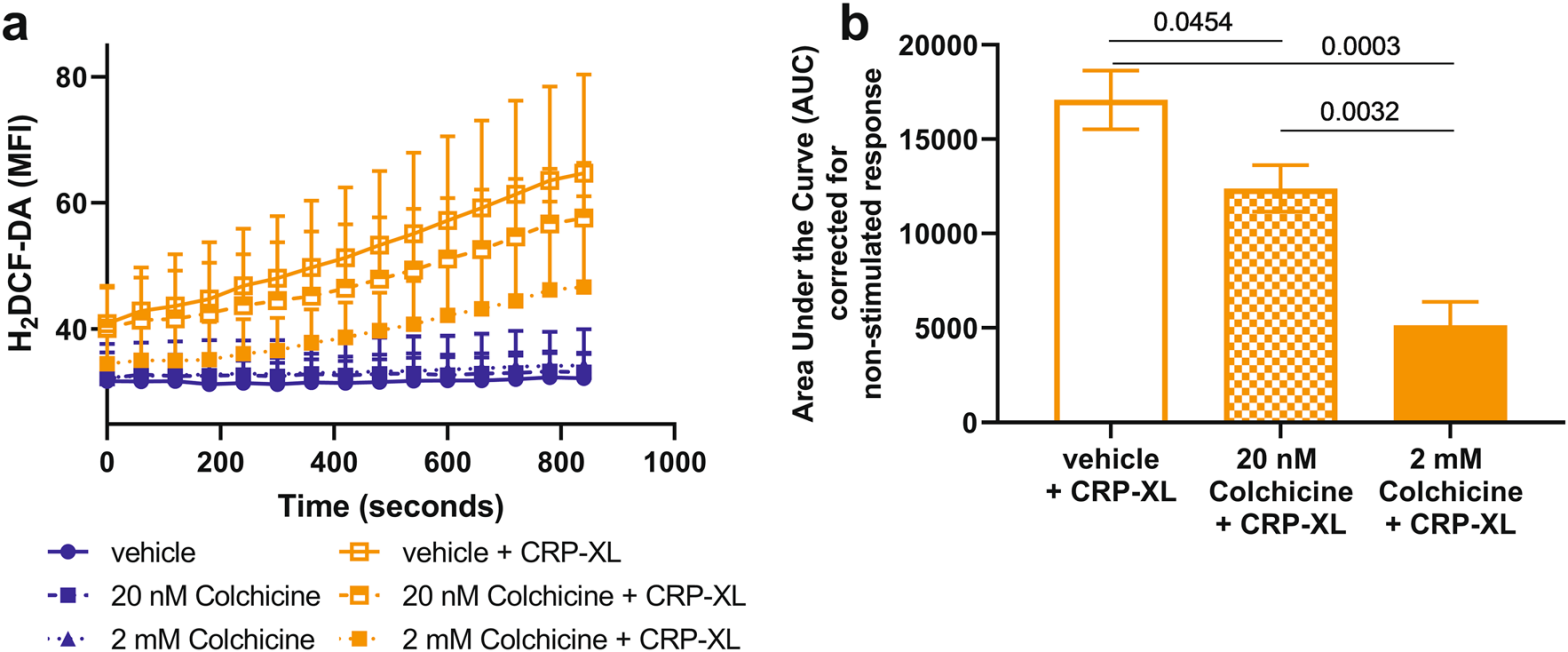
Colchicine inhibits ROS generation in response to GPVI stimulation with CRP-XL. Citrated PRP was used to assess the effect of colchicine on ROS generation, by flow cytometry with H_2_DCF-DA, in response to stimulation of the GPVI receptor with CRP-XL (2 µg/mL) or no agonist control. (**a**) ROS generation was reduced in platelets pre-incubated with colchicine in a concentration dependent manner in response to CRP-XL, no difference was observed between the no agonist controls. (**b**) ROS generation was significantly decreased by 20 nM (p = 0.045, n = 5) and 2 mM colchicine (p = 0.0003)—area under the curve from (**a**) corrected for the non-stimulated response. Statistical significance determined by Students *t* test; data presented as mean ± SD.

Use of the ROS dye 2′,7′-Dichlorofluorescein diacetate (H_2_DCF-DA) allows measurement of ROS generation in platelets by flow cytometry in response to agonist stimulation^42^. To further explore the effect of colchicine on the collagen receptor/GPVI pathway and the potential contribution of ROS to the colchicine-dependent reduction in platelet aggregation in response to collagen, platelets were stimulated with collagen-related peptide (CRP-XL) in PRP in the presence of H_2_DCF-DA, after preincubation with vehicle or colchicine (20 nM and 2 mM). Both low and high concentrations of colchicine significantly reduced ROS generation (p = 0.045 and p = 0.0003 respectively, n = 5) in platelets in response to stimulation of the GPVI receptor with CRP-XL (Fig. 3). ROS generation in response to ADP stimulation was not examined as previous studies have demonstrated that ADP stimulation of platelets under these conditions does not lead to an increase in ROS generation^42^.

### Colchicine does not cause shedding of the GPVI receptor

**Figure 4:**
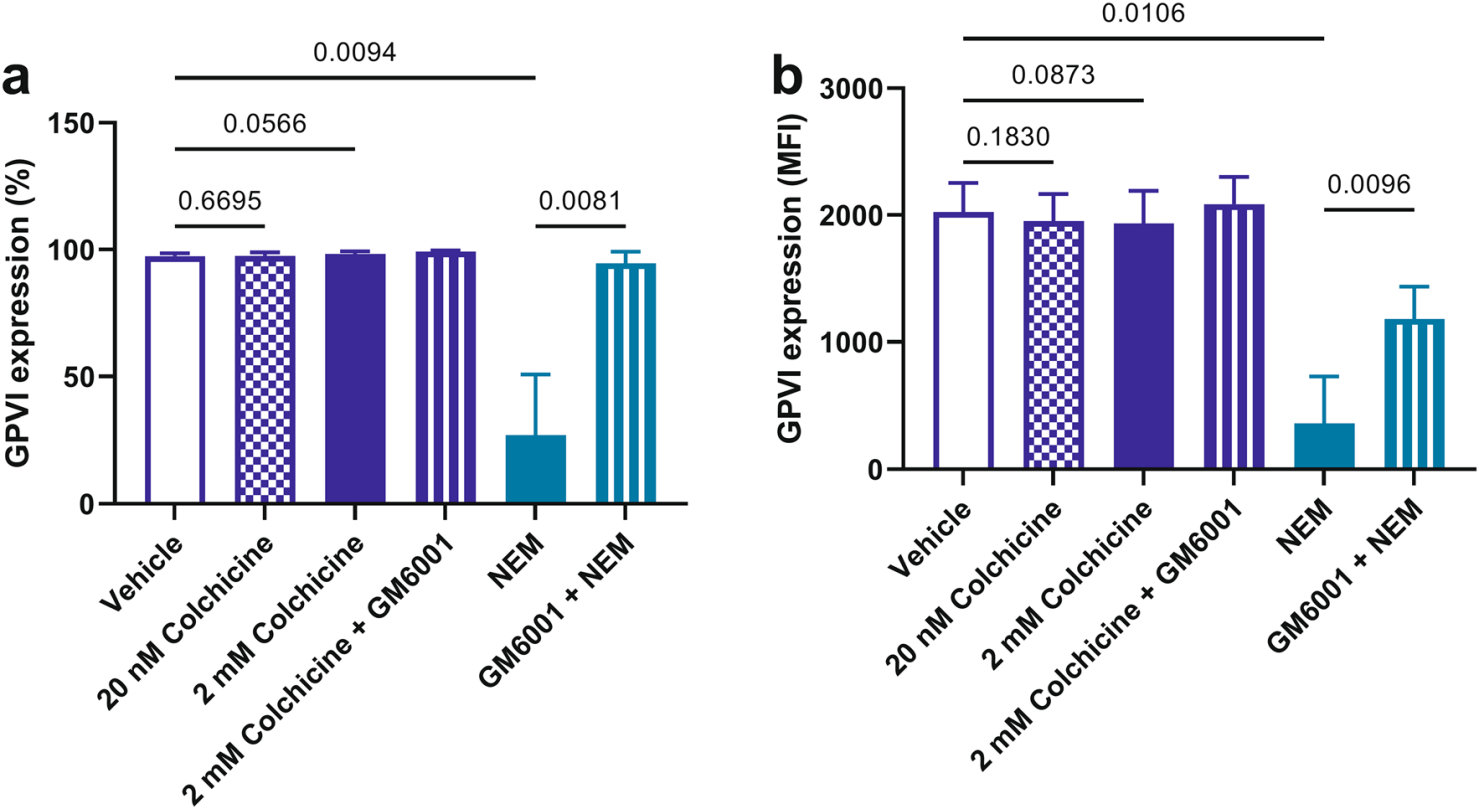
Colchicine does not cause GPVI shedding. Colchicine does not change platelet GPVI expression in PRP as assessed by %GPVI expression (**a**) or GPVI mean fluorescence intensity (**b**). GPVI was shed in response to the positive control (NEM, p = 0.009 for % and p = 0.01 for MFI) and rescued with GM6001 (a broad-spectrum metalloproteinase inhibitor, p = 0.008 for % and p = 0.01 for MFI). Statistical significance was determined by Students paired *t* test; data presented as mean ± SD; n = 4.

GPVI receptor expression can be assessed by flow cytometry. To determine if colchicine influenced GPVI receptor expression we assessed GPVI expression in all colchicine conditions (20 nM and 2 mM) as well as the vehicle control with a GPVI-AF488 (1G5) monoclonal antibody. To show that GPVI could be shed we included *N*-Ethylmaleimide (NEM) which is known to result in shedding of GPVI from human platelets as well as GM6001 (a broad spectrum metalloproteinase inhibitor) to see if there was a metalloproteinase effect of colchicine^43^. Colchicine did not lead to shedding of the GPVI receptor, and the addition of the NEM demonstrated that the platelets were capable of shedding GPVI (p = 0.009 for percentage expression and p = 0.01 for MFI expression) (Fig. 4).

### Colchicine does not suppress ADP-induced calcium flux

**Figure 5:**
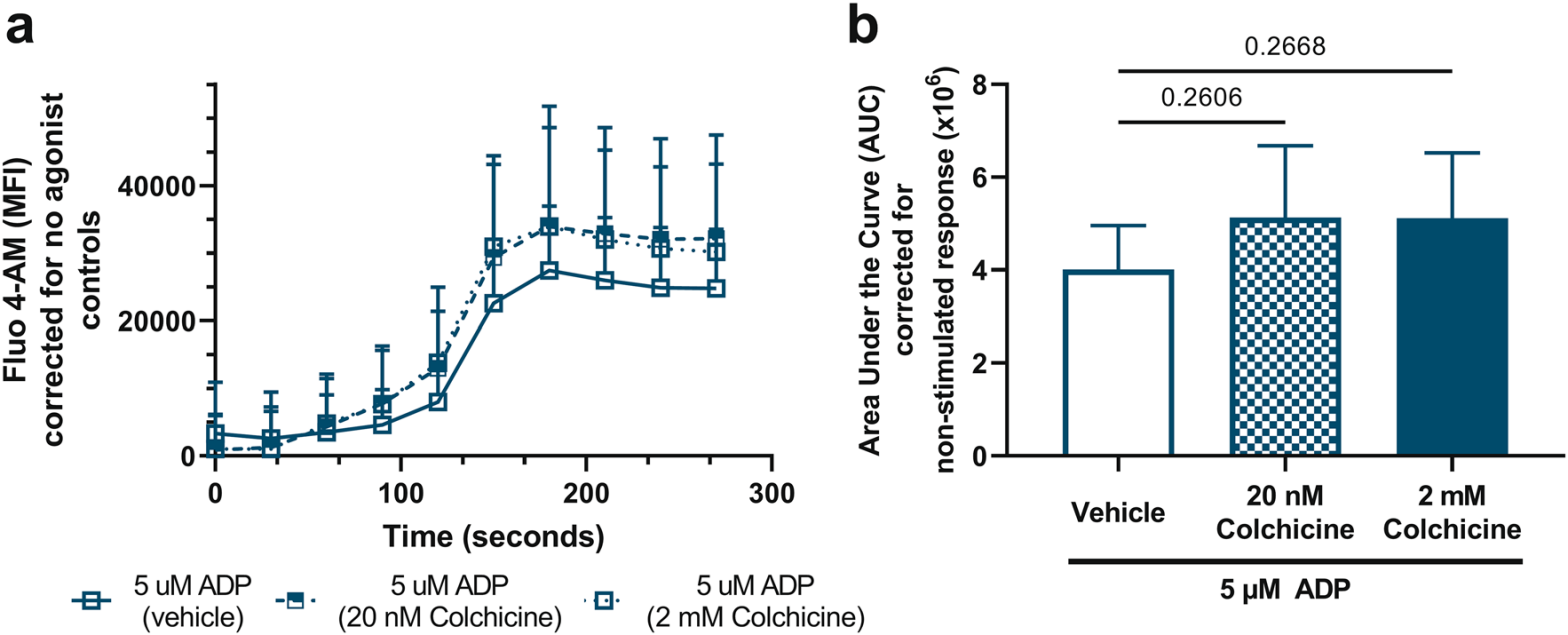
Colchicine does not alter ADP-induced calcium flux. Citrated PRP was used to assess the effect of colchicine on ADP-induced calcium flux by flow cytometry with Fluo-4 AM. (**a**) Calcium flux was not significantly altered in platelets pre-incubated with colchicine and stimulated with ADP (5 µM). Curves are corrected for the non-stimulated response. (**b**) Calcium flux was not significantly altered by 20 nM (p = 0.26) and 2 mM colchicine (p = 0.24)—area under the curve from (**a**). Statistical significance determined by Students *t* test; data presented as mean ± SD, n = 4 donors. The average of duplicate curves was used for each donor.

Use of Fluo 4-AM, a cell permeable fluorescent calcium indicator, allows measurement of calcium flux in platelets by flow cytometry in response to agonist. To determine if the effect of 2 mM colchicine on ADP stimulated aggregation was dependent on changes in calcium flux, we examined kinetic calcium flux changes after preincubation with vehicle control or colchicine. Neither concentration of colchicine (20 nM and 2 mM, n = 4) changed platelet calcium flux in response to ADP stimulation (Fig. 5).

### Effects of colchicine on platelet degranulation and GPIIb/IIIa conformational activation

**Figure 6:**
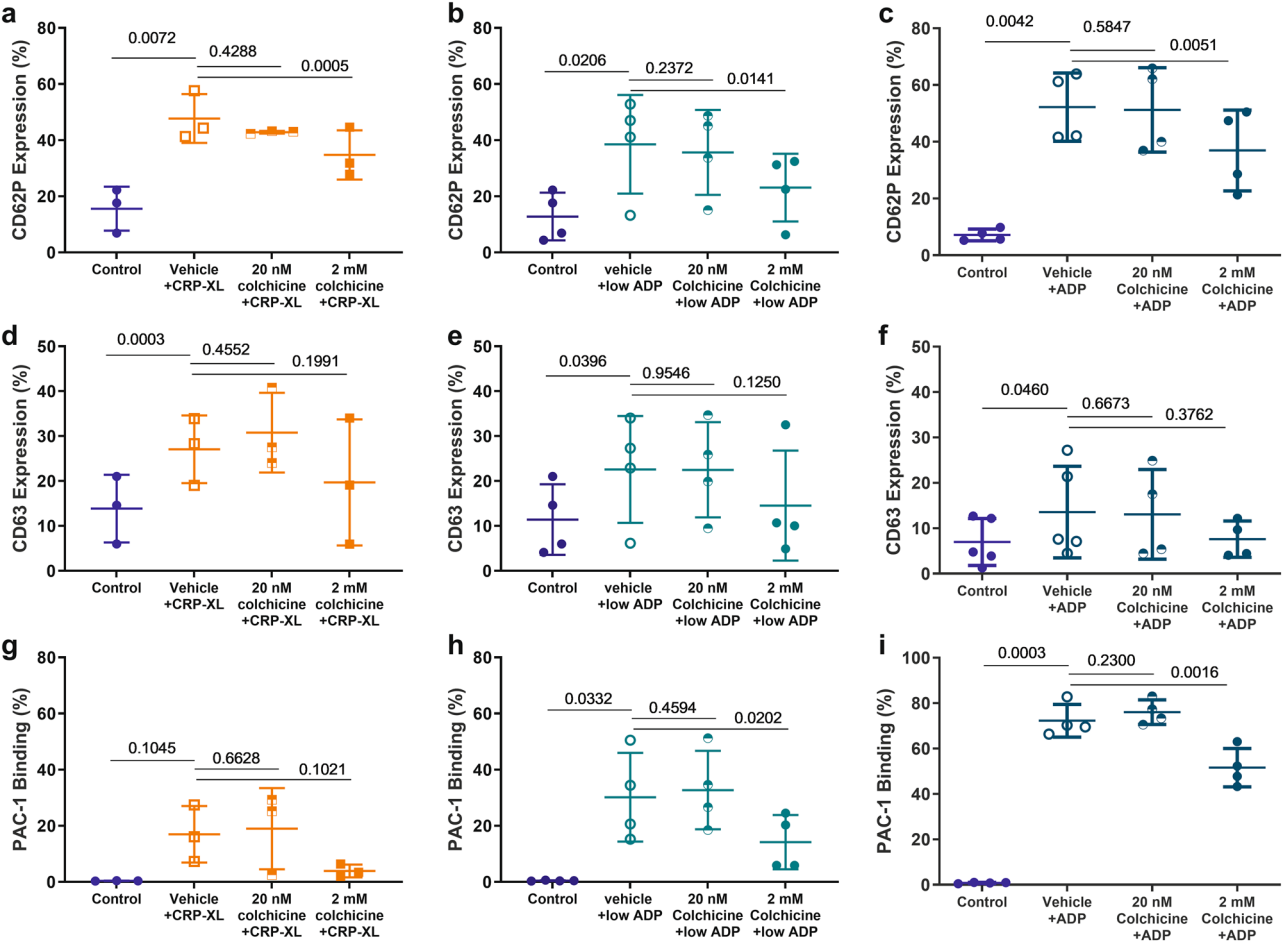
Effect of colchicine on P-selectin, CD63 expression and conformational change of GPIIb/IIIa. Flow cytometry was performed to assess the cell surface expression of P-selectin/CD62P, CD63 and conformational change of GPIIb/IIIa in response to stimulation with 0.2 µg/mL CRP-XL, 0.5 µM ADP or 2 µM ADP after preincubation with vehicle or colchicine (20 nM and 2 mM). A significant reduction in CD62P expression was only observed in those samples pre-incubated with high concentrations of colchicine [(**a**) CRP-XL, p = 0.0005; (**b**) 0.5 µM ADP, p = 0.014 and (**c**) 2 µM ADP, p = 0.005]. There was no significant difference in CD63 expression in response to colchicine under any of the conditions examined [(**d**) CRP-XL, (**e**) 0.5 µM ADP and (**f**) 2 µM ADP]. A significant reduction was only observed in GPIIb/IIIa conformational change (PAC-1 binding) in those samples pre-incubated with high concentrations of colchicine and stimulated with ADP, no change was observed in platelets stimulated with CRP-XL (**g**) [(**h**) 0.5 µM ADP, p = 0.02 and (**i**) 2 µM ADP, p = 0.002]. Statistical significance was determined by comparing conditions with the vehicle control using either paired *t* test or Wilcoxon matched pair rank test as appropriate. Data presented as mean ± SD, n = 3–4.

We next investigated the effects of colchicine on specific components of platelet activation in response to CRP-XL (0.5 µg/mL) and ADP (0.5 and 2 µM) using PRP from healthy volunteers. Flow cytometry was used to analyse the expression of CD62P (alpha granules), CD63 (dense and lysosomal granules) and PAC-1 binding (conformational activation of GPIIb/IIIa). At high concentrations, but not at typical plasma concentrations, colchicine significantly inhibited the upregulation of CD62P in response to CRP-XL (p = 0.0005, Fig. 6a) and ADP (p = 0.014 and 0.0051, 0.5 µM and 2 µM ADP respectively, Fig. 6b,c). No significant changes with colchicine incubation were observed in CD63 expression in response to stimulation of the collagen receptor GPVI, or P2Y_12_ receptors (Fig. 6d–f). Conformational change of GPIIb/IIIa was only observed after stimulation with ADP (both 0.5 and 2 µM) and not in response to CRP-XL (Fig. 6g), and its stimulation with ADP was inhibited only with 2 mM colchicine (Fig. 6h,i). Although the response to CRP-XL was not significant there was a trend towards an increase with stimulation and a decrease in the presence of colchicine.

### Microtubule depolymerisation-inhibitory concentrations of colchicine inhibit the formation of procoagulant platelets

**Figure 7:**
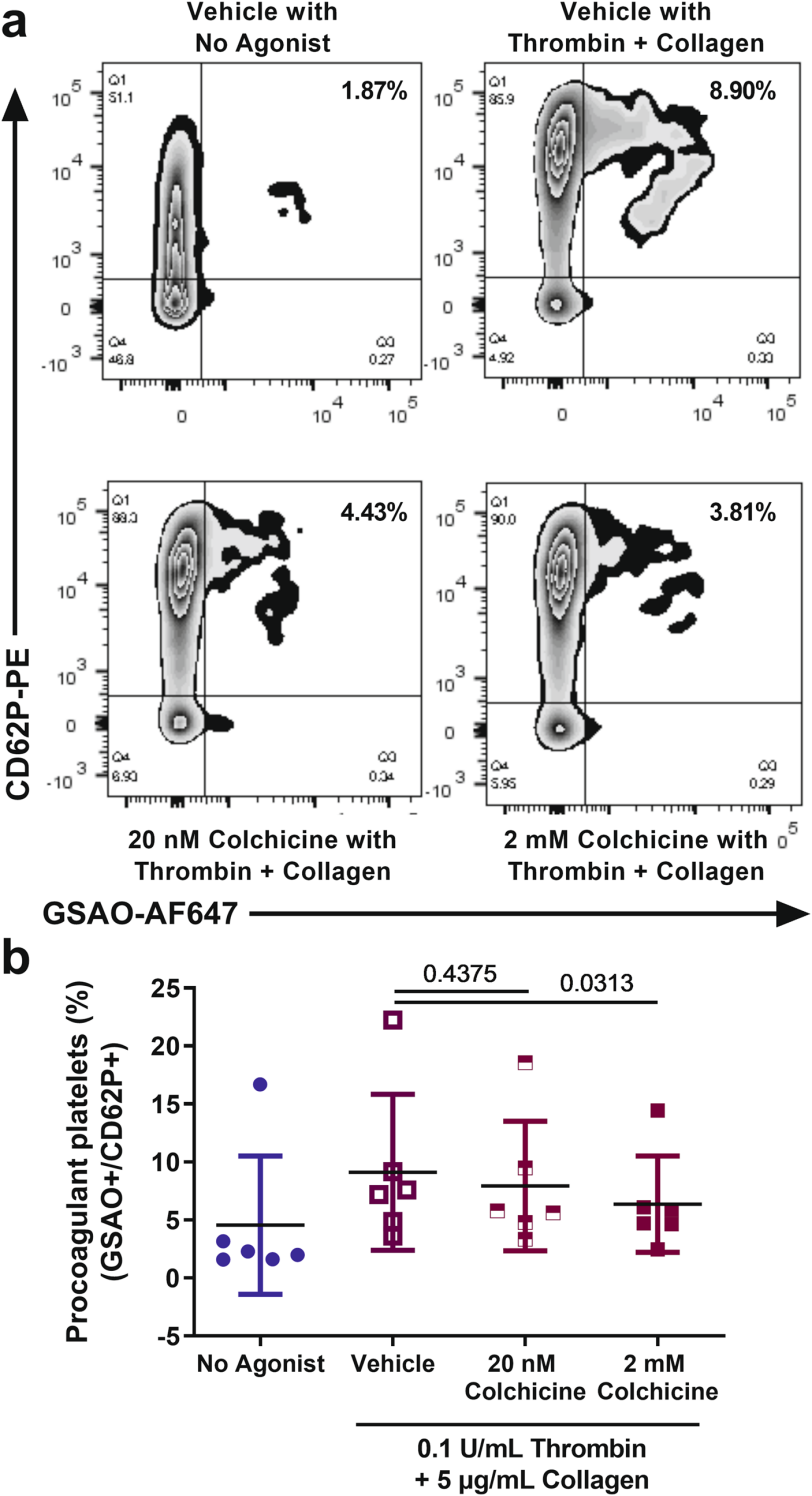
Colchicine inhibits the formation of procoagulant platelets. Washed platelets were subjected to dual stimulation with thrombin and collagen in the presence of vehicle, 20 nM or 2 mM colchicine, and the formation of procoagulant (GSAO^+^/CD62P^+^) platelets determined with flow cytometry. (**a**) Representative zebra plots showing procoagulant platelets (GSAO^+^/CD62P^+^, top right quadrant) at rest or after dual stimulation with thrombin and collagen with vehicle, 20 nM or 2 mM colchicine pre-incubation. (**b**) Percentage procoagulant platelets at rest, and after stimulation in the vehicle control, 20 nM and 2 mM colchicine. Mean ± SD, n = 6, Wilcoxon test vs vehicle control.

The capacity of CD62P positive platelets to bind GSAO after dual stimulation with thrombin and collagen has been characterised as a marker of platelet procoagulant status^33,34^. Washed platelets were exposed to 0.1 U/mL thrombin and 5 µg/mL collagen after preincubation with vehicle control or colchicine. The agonist stimulation caused procoagulant platelet formation and preincubation with 2 mM colchicine (and not 20 nM colchicine) significantly reduced this (n = 6, p = 0.031, Fig. 7). A variable degree of colchicine-mediated platelet cytotoxicity has been reported previously^15,25^, but under our experimental conditions colchicine did not induce apoptosis of the washed platelets (GSAO^+^/P-selectin^−^ platelets: 0.64 ± 0.45%, 0.59 ± 0.43%, 0.61 ± 0.26%—mean ± SD (n = 6) for vehicle, 20 nM colchicine and 2 mM colchicine respectively). The reduction in procoagulant platelets mediated by colchicine is therefore unlikely to be explained by loss of platelets related to cytotoxicity.

## Discussion

In this study we investigated the effects of circulating therapeutic concentrations (20 nM) and microtubule depolymerisation-inhibitory concentrations (2 mM) of colchicine on platelet activation. We identify that therapeutic concentrations of colchicine inhibit collagen-mediated platelet activation, specifically downstream GPVI receptor/pathway mediated ROS generation and reduce platelet aggregation independent of GPVI receptor shedding. At microtubule depolymerisation-inhibitory concentrations there is also inhibition of conformational activation of GPIIb/IIIa, granule release and procoagulant platelet formation. As colchicine did not affect calcium flux or platelet aggregation in response to stimulation of the P2Y_12_ receptor with ADP, these results indicate pathway specific mechanisms of action of colchicine on platelets and give new insight to the beneficial effect of colchicine in the treatment of cardiovascular disease.

GPVI has been considered a potential antithrombotic target for thrombotic and inflammatory diseases such as CAD. Stimulation of the GPVI receptor is distinct from the other platelet activation pathways traditionally targeted by antiplatelet therapy. GPVI inhibitors, including antibodies and GPVI-Fc fusion proteins such as “Revacept”, have demonstrated effectiveness as antiplatelet agents that do not lead to bleeding complications and can assist with disaggregation of thrombi^41,44^. Our study indicates that colchicine targets platelet aggregation and platelet activation (ROS generation) through the GPVI pathway, thereby providing an alternate treatment option that is also known for its anti-inflammatory effects. Colchicine is under investigation for this purpose^9,10^; we can now propose GPVI as a potential mechanism of action.

Previous studies^27^ observed a decrease in GPIIb/IIIa conformational change in response to epinephrine after in vivo treatment with colchicine but not a corresponding decrease in ADP- and epinephrine induced platelet aggregation. However, this finding does not preclude the possibility of an effect on collagen-induced aggregation. The same study did note a trend towards decreased platelet adherence on collagen after in vivo colchicine treatment and subsequent stimulation, further suggesting a potential effect of colchicine on the collagen receptor pathways and a potential effect on GPIIb/IIIa conformational change. Others have demonstrated an effect of in vitro colchicine on collagen stimulated aggregation, however, therapeutic levels of colchicine have not been examined^25,28^. In our study, the reduction in whole blood and PRP platelet aggregation with low concentrations of colchicine in vitro in response to collagen stimulation but not ADP stimulation suggests a relatively specific collagen receptor effect. Low concentrations of colchicine reduced ROS generation in response to CRP-XL independent of GPVI receptor shedding, as colchicine does not affect GPVI receptor expression. ROS generation is upstream of intracellular calcium flux which leads to platelet activation and GPIIb/IIIa conformational change^45^. The GPIIb/IIIa receptor and its conformational change are critical for platelet aggregation^46–48^, and GPIIb/IIIa receptor inhibitors reduce the risk of thrombotic events in patients with acute coronary syndromes undergoing coronary intervention (reviewed in^49–51^), however, an increased risk of bleeding is associated.

Previously, in vivo treatment with colchicine in healthy controls led to the reduction of platelet P-selectin expression, platelet-neutrophil aggregates and GPIIb/IIIa conformational change 2 h after receiving colchicine, with the GPIIb/IIIa effect lasting up to 24 h after dosing^27^. Interestingly, in our study, high concentrations of colchicine also led to a decrease in P-selectin expression and a reduction in GPIIb/IIIa conformational change in response to stimulation with ADP and CRP-XL. GPIIb/IIIa inhibitors are thought to affect platelet aggregation and activation via distinct actions, since addition of GPIIb/IIIa inhibitors in an in vitro system leads to a reduction in PAC-1 binding but has no effect on P-selectin expression in the presence of thrombin^52^. Therefore, it is possible that colchicine inhibits platelets by GPIIb/IIIa-related and unrelated pathways. Despite seeing a clear effect of low colchicine concentrations on collagen-induced aggregation and ROS generation, we did not observe a corresponding significant decrease in GPIIb/IIIa conformational change, this may be due to a higher concentration of colchicine being required in vitro*.*

Colchicine is known to accumulate within other blood cells specifically erythrocytes and to a lesser extent leukocytes^53^. In addition, it has been observed in vitro that colchicine binds to both plasma proteins and the platelet membrane, possibly altering the available colchicine concentration^22^, which may mean that the concentrations of colchicine available to platelets is substantially higher than the peak plasma concentrations (range 10–21 nM)^12,13^ would suggest. The amount of colchicine absorbed by the blood cells and found within the plasma can be quite variable between donors due to differences in bioavailability^53^. Therefore, the effects seen in an in vitro setting with higher concentrations of colchicine may be very relevant to effects that may be occurring in vivo*.*

Low concentrations of colchicine seemed to have less of an effect on the platelet response to stimulation of P2Y_12_ receptors with ADP, with neither platelet aggregation nor calcium flux being significantly altered by colchicine under these conditions. This is in agreement with earlier studies that demonstrated that low concentrations of colchicine have only minor effects on platelet aggregation and higher concentrations are required to see an effect^25,27,28^. That we have seen a significant effect of low concentrations of colchicine on the platelet response to collagen but not ADP suggests that the mechanism/s of action of colchicine may have some specificity in regard to the effect on the platelet pathways.

GSAO^+^/P-selectin^+^ platelets are a subset of stimulated platelets that actively support coagulation by providing a procoagulant surface during thrombosis^33,54^. In our study, high concentrations of colchicine suppressed the formation of procoagulant (GSAO^+^/P-selectin^+^) platelets in response to thrombin + collagen stimulation, without inhibiting total P-selectin expression in platelets. This implies a relatively specific inhibition of procoagulant platelet formation under these conditions. Collagen and thrombin are both demonstrable in atherosclerotic plaque and suppressing the formation of procoagulant platelets in response to these agonists may be particularly relevant in the context of coronary events, an area in which colchicine has shown recent promise^5,9,11^. Targeting the procoagulant platelet in atherothrombosis is attractive as it would reduce fibrin formation at the site of platelet aggregation without affecting fibrin formation at the endothelial surface, thus theoretically limiting bleeding complications^55^. Currently, there are very few therapeutic interventions that demonstrate reduction in procoagulant platelet formation. Acetazolamide, targeting aquaporin formation, and potassium and calcium channel inhibitors inhibit platelet shape change and platelet phosphatidylserine exposure during this process^56–58^. Whether clinical treatment with colchicine affects procoagulant platelet formation in vivo is currently unknown. Colchicine binds platelet microtubules^25^, and perturbed microtubule rearrangement could affect platelet shape change in the process of procoagulant platelet formation, but the mechanisms by which colchicine inhibits procoagulant platelet formation in vitro require further investigation.

In general terms, the effects of colchicine on platelets may involve either microtubule disassembly or direct action on the platelet membrane. The lack of apparent influx of colchicine into platelets under conditions when platelet aggregation was inhibited^22^ suggests actions on the platelet plasma membrane. However, most reduction in platelet aggregation and activation previously has been demonstrated under conditions when there is also apparent microtubule disassembly^14,15^. Concentrations of colchicine capable of disrupting platelet aggregation have also been shown to inhibit internal rearrangement of platelet organelles and granules, which generally occurs after activation prior to platelet aggregate formation^23^. In the present study, we observed a decrease in aggregation with colchicine levels that have not yet been shown to affect the microtubule structure.

There are several limitations of this study which should be considered. A direct comparison with in vivo colchicine use would have been ideal since a higher concentration of colchicine was required in vitro to see changes in GPIIb/IIIa conformational change that have been published in vivo although with other agonists. A high concentration of 2 mM was used as a positive control for microtubule de-polymerization, but it is unclear what concentration of colchicine platelets are exposed to in vivo. While 20 nM colchicine is closer to measured peak plasma concentrations, the addition of 20 nM colchicine does not account for the sequestering of colchicine by erythrocytes and leukocytes or the known interactions with albumin. Therefore, 20 nM colchicine may be an underestimate of the actual concentrations of colchicine that platelets may be exposed to. Only healthy donors ranging from young to middle age were recruited for this study and whether platelets from older cohorts behave differently is unclear.

In conclusion, at low concentrations equivalent to those found in plasma after therapeutic treatment, colchicine modulated platelet activation/signalling pathways, causing a reduction in platelet aggregation and ROS generation, in a collagen receptor-specific manner. At high concentrations, colchicine inhibited platelet granule release in response to both collagen and ADP, as indicated by reduced P-selectin expression, as well as inhibiting GPIIb/IIIa conformational change and procoagulant platelet formation. These results provide insight into potential mechanisms for the protective effect of colchicine in the prevention of coronary events, additional to those facilitated by leukocyte-mediated inflammation.

## Methods

### Reagents

Pre-conjugated antibodies were from BD Bioscience (Franklin Lakes, NJ, USA) (CD41a, BV510, HIP8; CD61, PerCP-Cy5.5, VI-PL2; CD62P, PE, AK-4; CD63, PE, H5C6; GPIIb/IIIa conformational change, FITC, PAC-1; and isotype controls—IgG, PE, MOPC-21; IgM, FITC, GI55-228). GPVI AF488 (1G5) kindly donated by Prof Elizabeth Gardiner (ANU). Reagents included adenosine diphosphate (ADP), collagen (Helena Laboratories, Mt Waverly, VIC, Australia), colchicine, thrombin (bovine plasma), Gly-Pro-Arg-Pro amide (GPRP; dissolved in Hank’s balanced salt solution, HBSS, pH 7.35, at 10 mM and stored at − 20 °C), prostaglandin E1 (PGE1), N-Ethylmaleimide (NEM, dissolved in methanol) and GM6001 (a broad spectrum metalloproteinase inhibitor) (Sigma-Aldrich, St Louis, MO, USA), collagen related peptide (CRP-XL) (Auspep, Tullamarine, VIC, Australia) and equine tendon collagen type 1, Collagen Reagens HORM (Takeda, Osaka, Osaka Prefecture, Japan). Buffers included Dulbecco’s modified phosphate-buffered saline (DPBS), (Invitrogen, Carlsbad, CA, USA), paraformaldehyde 16% solution (ProSciTech, Kirwan, QLD, Australia), Hanks’ balanced salt solution (HBSS; Life Technologies, Carlsbad, CA, USA) and 0.9% sodium chloride solution (Baxter, Deerfield, IL, USA). Other reagents included PamFix (Platelet Solutions, UK); GSAO conjugated with Alexa Fluor 647 [4-(*N*-(*S*-glutathionylacetyl) amino) phenylarsonous acid]^33^; GSCA [4-(*N*-(*S*-glutathionylacetyl) amino) benzoic acid] conjugated with Alexa Fluor 647 and H_2_DCF-DA (Sigma).

### Blood collection

Ethics approval for this study was gained through the institutional human research ethics committee at Concord Repatriation General Hospital, Australia (HREC/15/CRGH/54). Written informed consent was obtained from all subjects in accordance with requirements of the ethics committee and the investigation conformed to the principles outlined in the Declaration of Helsinki.

Blood was collected from healthy volunteers as previously described^59^. Hirudin blood tubes (Multiplate, Verum Diagnostica GmbH; Munich, Germany) were used for platelet aggregometry and platelet activation studies, citrate blood collection tubes (BD Biosciences) were used for assessment of ROS generation and calcium flux, and ACD blood collection tubes (BD Biosciences) were used for washed platelet isolation for procoagulant platelet formation.

### Platelet Isolation

*Platelet-rich plasma* (PRP) was isolated by centrifuging hirudinised or citrated blood at 200*g* for 10 min. Hirudinised PRP was used for examining platelet aggregation, platelet granule release (CD62P and CD63) and conformational change of GPIIb/IIIa. Citrated PRP was used for examining ROS generation and calcium flux.

*Washed platelets* were isolated from ACD anticoagulated blood by first centrifuging at 200*g* for 20 min to isolate the PRP. The PRP was collected and mixed in a 1:1 ratio with DPBS containing 1 µM prostaglandin E1 (PGE1), then centrifuged at 100*g* for 15 min. The supernatant was collected and centrifuged at 800*g* for 20 min to pellet the platelets before being gently washed with DPBS and re-centrifuged at 800*g* for 15 min. The washed pellet was gently resuspended in phosphate-free Tyrode’s buffer (138 mM NaCl, 2.6 mM KCl, 12 mM NaHCO_3_, 5.5 mM glucose, 1.8 mM CaCl_2_, 0.49 mM MgCl_2_, pH 7.4) and adjusted to 2–3 × 10^8^ platelets/mL after automated full blood count analysis. Washed platelets were used to examine the effect of colchicine on procoagulant platelet formation.

### Colchicine incubations

PRP, washed platelets or whole blood was incubated with the desired final concentration of colchicine (20 nM or 2 mM) for 30 min at 37 °C on a rotary tube mixer using non-pyrogenic endotoxin-free water as the vehicle control.

### Multiplate aggregometry

To determine if colchicine affects platelet aggregation in whole blood or PRP, Multiplate aggregometry was performed as per the manufacturer’s instructions using 6.7 µM ADP or 3 µg/mL collagen (all final concentrations) after preincubation with either vehicle control or colchicine (20 nM or 2 mM). Aggregometry was performed on both hirudinised whole blood (vehicle and 20 nM only) and hirudinised PRP. Results are expressed as area under the curve (AUC) for aggregation and velocity (AU/min) for the rate of aggregation that occurs over 6 min.

### Flow cytometry

#### Platelet intracellular reactive oxygen species (ROS)

To determine if colchicine affects collagen receptor signalling in a ROS-dependent manner, platelet intracellular ROS levels were measured by flow cytometry with the ROS dye, 2′,7′-Dichlorofluorescein diacetate (H_2_DCF-DA)^42^. Briefly, PRP (citrate) was incubated with H_2_DCF-DA (10 µM final concentration, Sigma) for 30 min at 37 °C in the dark. The dye loaded PRP samples were incubated with vehicle control or 20 nM colchicine for 30 min at 37 °C as described above. Vehicle or colchicine containing samples were stimulated with 2 µg/mL CRP-XL for 1 min and then diluted tenfold with calcium free Tyrode’s buffer (containing 0.1% BSA, 10 µM H_2_DCF-DA). Samples were run on a BD LSRFortessa X-20 with the dye positive platelets being examined over time (15 min). Analysis was performed on FlowJo X.0.7 (FlowJo, LLC, Ashland, OR, USA) with the “Kinetics” function, assessing the geometric mean expression of H_2_DCF-DA over time in 30 s intervals.

#### Calcium flux

To determine if colchicine affects ADP-stimulated calcium flux, intracellular platelet calcium flux was assessed by flow cytometry using the calcium indicator dye Fluo-4 AM^60^. PRP (citrate) was incubated with Fluo-4 AM (5 µM final concentration, abcam, Cambridge, UK) in the presence of calcium and magnesium free Tyrode’s buffer for 15 min at 37 °C. An aliquot was incubated with CD41a BV510 (BD Biosciences) and GPRP (Sigma) for 15 min at 37 °C. This was then diluted in Tyrode’s buffer containing CaCl_2_ and MgCl_2_. The sample was stimulated with ADP at 30 s with a final concentration of 5 µM, data was collected for 5 min and the stimulations were performed in duplicate for each donor. A no agonist control was also run. Flow cytometry was performed on Beckman Coulter CytoFLEX S Flow Cytometer and analysis was performed on FlowJo X.0.7 with the “Kinetics” function, assessing the geometric mean expression of Fluo-4 AM in the CD41a positive population over time in 30 s intervals.

#### Platelet surface marker expression

To determine if colchicine affects platelet activation (CD62P, CD63 expression) or conformational change of GPIIb/IIIa (PAC-1 binding), flow cytometry was performed under non-stimulated or agonist stimulated conditions after incubation with vehicle control or colchicine (20 nM and 2 mM). Stimulation was with CRP-XL (0.2 µg/mL) or ADP (0.5 µM and 2 µM) in hirudinised PRP. DPBS, agonist and PRP were gently mixed and then added directly to the FACS tubes containing the appropriate antibodies and left to incubate for 30 min at room temperature in the dark. These were then fixed with 0.16% paraformaldehyde solution in saline, run on a BD FACSCalibur™ or BD LSRFortessa X-20 and analysed by FlowJo X.0.7. Isotype controls were used to establish 0.5% background expression in the CD61 positive platelet population. Positive percentage expression or binding was then determined.

#### GPVI receptor expression

To determine if colchicine causes GPVI receptor shedding, flow cytometry was performed under non-stimulated conditions. Citrated PRP was incubated with colchicine as indicated above. Where GM6001 was used the PRP was preincubated with 250 µM GM6001 for 15 min at room temperature prior to the colchicine incubation to inhibit metalloproteinase induced shedding. The positive control for GPVI receptor shedding was 5 mM NEM, the incubation was performed concurrently with colchicine under colchicine incubation conditions. After the incubations were performed, PRP was incubated in the presence of GPVI-AF488 antibody, CD61 PerCP-Cy5.5 and DPBS. The samples were then fixed with 0.16% paraformaldehyde solution in saline, run on a BD LSRFortessa X-20 and analysed by FlowJo X.0.7. Positive percentage GPVI expression was determined by gating CD61 positive platelets on the positive peak from the NEM incubated sample. Geometric mean was used for the mean fluorescence intensity (MFI) result.

#### Procoagulant platelets

Following incubation with colchicine or vehicle control, procoagulant platelets were assessed as previously described^33^. Briefly, 15 µL of washed platelets (5 × 10^8^ platelets/mL) were diluted into HBSS buffer (pH 7.35 containing a final concentration of 2.5 mM GPRP and 2.5 mM CaCl_2_ in the end reaction mix). Platelets were stimulated with 0.1 U/mL thrombin and 5 µg/mL collagen for 10 min at room temperature. The reaction was stopped by dilution with HBSS and aliquots labelled for 15 min at room temperature with GSAO AF647 (1 µM) and CD62P PE. After labelling, cells were fixed with PamFix for 5 min. Fixed and stained cells were washed once before resuspension and analysis by flow cytometry on a BD FACSCanto™ II to measure the proportion of generation of procoagulant platelets. Procoagulant platelets were defined as those that co-stained with GSAO and P-selectin^33^.

#### Platelet apoptosis

To assess for toxicity from colchicine, washed human platelets were stimulated and labelled as above, and apoptotic platelets were defined as GSAO^+^/P-selectin^−^ platelets^33^.

### Statistical methods

All statistical analyses were performed using Prism version 8.2.0 (GraphPad Software, Inc., San Diego, CA). Normality of the data was assessed by using the D’Agnostino and Pearson test as well as the stem and leaf plots or boxplots. Continuous variables were presented as mean ± standard deviation (SD); n represents the number of experiments. Colchicine response to collagen in whole blood, platelet rich plasma and GPVI stimulation with CRP-XL was quantified by using the area under the curve (AUC). Data were tested for significance using the paired Student *t* test. All tests were two tailed and results with values of P < 0.05 were considered to be statistically significant.

## Supplementary Information

Supplementary Figure S1.

## Data Availability

All data generated or analysed during this study are included in this published article.

## References

[1] Deftereos S (2013). Colchicine and the heart: Pushing the envelope. J. Am. Coll. Cardiol..

[2] Imazio M (2015). Colchicine for pericarditis. Trends Cardiovasc. Med..

[3] Fiolet ATL (2020). Short-term effect of low-dose colchicine on inflammatory biomarkers, lipids, blood count and renal function in chronic coronary artery disease and elevated high-sensitivity C-reactive protein. PLoS One.

[4] Katsanos AH (2020). Colchicine for stroke prevention in patients with coronary artery disease: A systematic review and meta-analysis. Eur. J. Neurol..

[5] Nidorf SM, Eikelboom JW, Thompson PL (2014). Colchicine for secondary prevention of cardiovascular disease. Curr. Atheroscler. Rep..

[6] Papageorgiou N, Briasoulis A, Lazaros G, Imazio M, Tousoulis D (2017). Colchicine for prevention and treatment of cardiac diseases: A meta-analysis. Cardiovasc. Ther..

[7] Tan G-M, Yan BP (2017). What’s old is new again—A review of the current evidence of colchicine in cardiovascular medicine. Curr. Cardiol. Rev..

[8] Martinez GJ (2015). Colchicine acutely suppresses local cardiac production of inflammatory cytokines in patients with an acute coronary syndrome. J. Am. Heart Assoc..

[9] Nidorf SM, Eikelboom JW, Budgeon CA, Thompson PL (2013). Low-dose colchicine for secondary prevention of cardiovascular disease. J. Am. Coll. Cardiol..

[10] Nidorf SM (2019). The effect of low-dose colchicine in patients with stable coronary artery disease: The LoDoCo2 trial rationale, design, and baseline characteristics. Am. Heart J..

[11] Opstal TSJ (2020). Colchicine attenuates inflammation beyond the inflammasome in chronic coronary artery disease: A LoDoCo2 proteomic substudy. Circulation.

[12] Chappey ON (1993). Colchicine disposition in human leukocytes after single and multiple oral administration. Clin. Pharmacol. Ther..

[13] Ferron GM, Rochdi M, Jusko WJ, Scherrmann JM (1996). Oral absorption characteristics and pharmacokinetics of colchicine in healthy volunteers after single and multiple doses. J. Clin. Pharmacol..

[14] Bouaziz A (2007). Tyrosine phosphorylation/dephosphorylation balance is involved in thrombin-evoked microtubular reorganisation in human platelets. Thromb. Haemost..

[15] White JG (1968). Effects of colchicine and Vinca alkaloids on human platelets. I. Influence on platelet microtubules and contractile function. Am. J. Pathol..

[16] Martinon F, Petrilli V, Mayor A, Tardivel A, Tschopp J (2006). Gout-associated uric acid crystals activate the NALP3 inflammasome. Nature.

[17] Cronstein BN (1995). Colchicine alters the quantitative and qualitative display of selectins on endothelial cells and neutrophils. J. Clin. Investig..

[18] Korkmaz S (2011). Colchicine modulates oxidative stress in serum and neutrophil of patients with Behcet disease through regulation of Ca(2)(+) release and antioxidant system. J. Membr. Biol..

[19] Lebas H, Yahiaoui K, Martos R, Boulaftali Y (2019). Platelets are at the nexus of vascular diseases. Front. Cardiovasc. Med..

[20] Nording HM, Seizer P, Langer HF (2015). Platelets in inflammation and atherogenesis. Front. Immunol..

[21] Hardwick RA, Gritsman HN, Stromberg RR, Friedman LI (1983). The biochemical mechanisms of shear-induced platelet aggregation. Trans. Am. Soc. Artif. Intern. Organs.

[22] Ribbi-Jaffe A, Apitz-Castro R (1979). The effect of colchicine on human blood platelets under conditions of short-term incubation. Biochem. J..

[23] Sneddon JM (1971). Effect of mitosis inhibitors on blood platelet microtubules and aggregation. J. Physiol..

[24] Soppitt GD, Mitchell JR (1969). The effect of colchicine on human platelet behaviour. J. Atheroscler. Res..

[25] Menche D, Israel A, Karpatkin S (1980). Platelets and microtubules. Effect of colchicine and D2O on platelet aggregation and release induced by calcium ionophore A23187. J. Clin. Investig..

[26] White JG (1969). Effects of colchicine and vinca alkaloids on human platelets. 3. Influence on primary internal contraction and secondary aggregation. Am. J. Pathol..

[27] Shah B (2016). Effect of colchicine on platelet-platelet and platelet-leukocyte interactions: A pilot study in healthy subjects. Inflammation.

[28] Cimmino G (2018). Colchicine reduces platelet aggregation by modulating cytoskeleton rearrangement via inhibition of cofilin and LIM domain kinase 1. Vascul. Pharmacol..

[29] Cirillo P (2020). Effects of colchicine on platelet aggregation in patients on dual antiplatelet therapy with aspirin and clopidogrel. J. Thromb. Thrombolysis.

[30] Reddel CJ, Pennings GJ, Curnow JL, Chen VM, Kritharides L (2018). Procoagulant effects of low-level platelet activation and its inhibition by colchicine. Thromb. Haemost..

[31] Yong AS (2011). Intracoronary shear-related up-regulation of platelet P-selectin and platelet-monocyte aggregation despite the use of aspirin and clopidogrel. Blood.

[32] Springer TA, Zhu J, Xiao T (2008). Structural basis for distinctive recognition of fibrinogen gammaC peptide by the platelet integrin alphaIIbbeta3. J. Cell Biol..

[33] Hua VM (2015). Necrotic platelets provide a procoagulant surface during thrombosis. Blood.

[34] Pasalic L (2018). Novel assay demonstrates that coronary artery disease patients have heightened procoagulant platelet response. J. Thromb. Haemost..

[35] Ridker PM (2017). Antiinflammatory therapy with canakinumab for atherosclerotic disease. N. Engl. J. Med..

[36] Andrews RK, Arthur JF, Gardiner EE (2014). Targeting GPVI as a novel antithrombotic strategy. J. Blood Med..

[37] Mingant F (2018). Comparison of four methods to assess high-on platelet reactivity under P2Y12 receptor inhibitor. Platelets.

[38] Borst O, Gawaz M (2021). Glycoprotein VI—Novel target in antiplatelet medication. Pharmacol. Ther..

[39] Rayes J, Watson SP, Nieswandt B (2019). Functional significance of the platelet immune receptors GPVI and CLEC-2. J. Clin. Investig..

[40] Chatterjee M, Gawaz M (2017). Clinical significance of receptor shedding-platelet GPVI as an emerging diagnostic and therapeutic tool. Platelets.

[41] Ahmed MU (2020). Pharmacological blockade of glycoprotein VI promotes thrombus disaggregation in the absence of thrombin. Arterioscler. Thromb. Vasc. Biol..

[42] Arthur JF (2012). ITAM receptor-mediated generation of reactive oxygen species in human platelets occurs via Syk-dependent and Syk-independent pathways. J. Thromb. Haemost..

[43] Gardiner EE (2007). Controlled shedding of platelet glycoprotein (GP)VI and GPIb-IX-V by ADAM family metalloproteinases. J. Thromb. Haemost..

[44] Ungerer M (2013). The GPVI-Fc fusion protein Revacept reduces thrombus formation and improves vascular dysfunction in atherosclerosis without any impact on bleeding times. PLoS ONE.

[45] Suzuki-Inoue K (2004). Glycoproteins VI and Ib-IX-V stimulate tyrosine phosphorylation of tyrosine kinase Syk and phospholipase Cgamma2 at distinct sites. Biochem. J..

[46] Coller BS (1985). A new murine monoclonal antibody reports an activation-dependent change in the conformation and/or microenvironment of the platelet glycoprotein IIb/IIIa complex. J. Clin. Investig..

[47] Coller BS, Folts JD, Scudder LE, Smith SR (1986). Antithrombotic effect of a monoclonal antibody to the platelet glycoprotein IIb/IIIa receptor in an experimental animal model. Blood.

[48] Coller BS, Scudder LE (1985). Inhibition of dog platelet function by in vivo infusion of F(ab')2 fragments of a monoclonal antibody to the platelet glycoprotein IIb/IIIa receptor. Blood.

[49] Tcheng JE (1996). Glycoprotein IIb/IIIa receptor inhibitors: Putting the EPIC, IMPACT II, RESTORE, and EPILOG trials into perspective. Am. J. Cardiol..

[50] Kong DF, Califf RM (1999). Glycoprotein IIb/IIIa receptor antagonists in non-ST elevation acute coronary syndromes and percutaneous revascularisation: A review of trial reports. Drugs.

[51] Muniz-Lozano A, Rollini F, Franchi F, Angiolillo DJ (2013). Update on platelet glycoprotein IIb/IIIa inhibitors: Recommendations for clinical practice. Ther. Adv. Cardiovasc. Dis..

[52] Caron A, Theoret JF, Mousa SA, Merhi Y (2002). Anti-platelet effects of GPIIb/IIIa and P-selectin antagonism, platelet activation, and binding to neutrophils. J. Cardiovasc. Pharmacol..

[53] Sabouraud A, Chappey O, Dupin T, Scherrmann JM (1994). Binding of colchicine and thiocolchicoside to human serum proteins and blood cells. Int. J. Clin. Pharmacol. Ther..

[54] Hua VM, Chen VM (2015). Procoagulant platelets and the pathways leading to cell death. Semin. Thromb. Hemost..

[55] Jackson SP (2011). Arterial thrombosis–insidious, unpredictable and deadly. Nat. Med..

[56] Harper MT, Poole AW (2013). Chloride channels are necessary for full platelet phosphatidylserine exposure and procoagulant activity. Cell Death Dis..

[57] Wolfs JL (2006). Reversible inhibition of the platelet procoagulant response through manipulation of the Gardos channel. Blood.

[58] Agbani EO (2015). Coordinated membrane ballooning and procoagulant spreading in human platelets. Circulation.

[59] Pennings GJ, Yong AS, Kritharides L (2010). Expression of EMMPRIN (CD147) on circulating platelets in vivo. J. Thromb. Haemost..

[60] Pasalic L (2017). Flow cytometry protocols for assessment of platelet function in whole blood. Methods Mol. Biol..

