# Supplementary material for: Colchicine inhibits ROS generation in response to glycoprotein VI stimulation
Source: Sci Rep. 2021 Jun 7;11:11965. doi: 10.1038/s41598-021-91409-7 (PMC8184800; doi:10.1038/s41598-021-91409-7)
Supplement: Supplementary file 1 — Supplementary Figure S1. [file 41598_2021_91409_MOESM1_ESM.pdf]

Title: Colchicine inhibits ROS generation in response to glycoprotein VI stimulation

Authors: GJ Pennings*[1,4]; CJ Reddel[1,4]; M Traini[1,4]; H Campbell[1]; V Chen[1,2,4]; L Kritharides*[1,3,4]

Supplementary Data

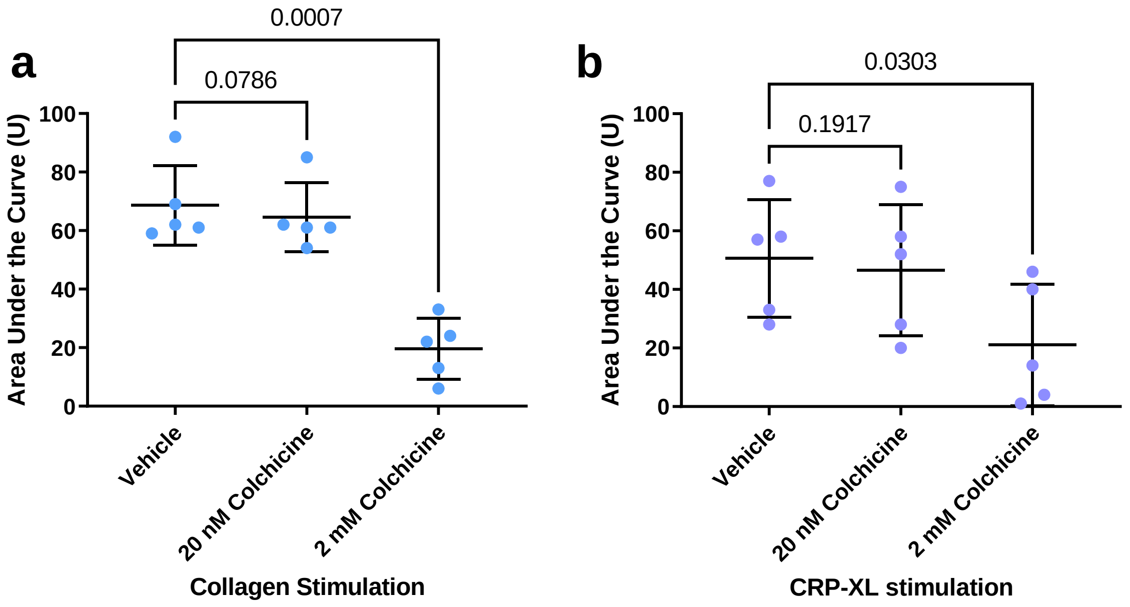

Supplementary Figure 1: Effect of collagen and CRP-XL on PRP aggregation performed in parallel. PRP aggregation induced by collagen (a) or CRP-XL (b) is decreased non-significantly by 20 nM colchicine but is strongly inhibited by 2 mM colchicine preincubation. Significance determined by students paired t-test, p<0.05 considered significant. Mean±SD, n=5.
